# The Effects of Guided Imagery on Women’s Health: A Systematic Review of Experimental Studies

**DOI:** 10.3390/ijerph23081066

**Published:** 2026-08-17

**Authors:** Şehma Şen, Duygu Gözen

**Affiliations:** 1 Nursing (English) Department, Istanbul Atlas University, İstanbul 34408, Türkiye; 2 Faculty of Nursing, Koç University, İstanbul 34010, Türkiye; dugozen@ku.edu.tr

**Keywords:** complementary therapies, guided imagery, mind–body therapies, nursing, systematic review, women’s health

## Abstract

**Highlights:**

**Public health relevance—How does this work relate to a public health issue?**

- Women naturally experience distinct biological, psychological, and sociocultural life-cycle transitions—such as pregnancy, childbirth, menopause, and gynecological cancers—that make them highly vulnerable to stress, anxiety, and psychosomatic symptoms.
- Modern public health requires the integration of accessible, patient-centered, and non-pharmacological interventions to holistically address the complex interplay between psychological well-being and physiological processes in women’s health.

**Public health significance—Why is this work of significance to public health?**

- This systematic review synthesizes scientific evidence from 29 experimental studies involving 1939 women, confirming that guided imagery significantly mitigates psychological distress (anxiety, stress, depression) and manages physical symptoms (pain, fatigue, and treatment side effects).
- The findings establish that guided imagery has evolved from a simple relaxation tool into a highly effective, low-cost, and non-invasive mind–body intervention applicable across diverse clinical areas, including oncology, perinatal care, and surgical settings.

**Public health implications—What are the key implications or messages for practitioners, policy makers and/or researchers in public health?**

- Health professionals, particularly nurses and midwives, should systematically integrate guided imagery into standard clinical protocols and perinatal care to enhance the birthing experience, reduce surgical fear, and improve patient satisfaction.
- Policy makers and healthcare institutions should support evidence-based training and resources for mind–body therapies, bridging the gap between clinical evidence and practice to establish guided imagery as a standard, affordable component of women’s holistic health services.

**Abstract:**

Background: Guided imagery (GI) is a mind–body intervention that uses sensory visualizations to promote relaxation and healing. While being utilized in various clinical settings, its specific cumulative impact on women’s health across different life stages remains to be comprehensively synthesized. This systematic review aims to evaluate the effectiveness of GI on psychological and physiological outcomes in women’s health, including gynecologic/breast cancer, pregnancy, childbirth, and menopause. Methods: A systematic search was conducted across PubMed, Scopus, Web of Science, and CINAHL databases for studies published between 2014 and 2024. The search terms included keywords related to “guided imagery,” “women’s health,” and “experimental studies”. We included randomized controlled trials and quasi-experimental studies focusing on GI interventions in women. Data were extracted on study characteristics, participant demographics, intervention details, and outcomes. The methodological quality was assessed using the JBI Critical Appraisal Tools. Results: A total of 29 studies (27 RCTs, 2 quasi-experimental) involving 1939 women were included. GI was found to significantly reduce anxiety, stress, depression, pain, and fatigue. It also improved quality of life and the overall labor experience during childbirth. The synthesis revealed that GI has evolved from a simple relaxation tool to a holistic mind–body intervention. Conclusions: Guided imagery is an effective, non-invasive, and low-cost complementary intervention that improves various psychological and physiological health outcomes in women. It should be integrated into clinical practice as part of patient-centered, holistic nursing care.

## 1. Introduction

Women’s health is a multidimensional phenomenon shaped not only by biological differences but also by sociocultural roles, hormonal variations, and life-cycle transitions [1,2]. This complexity is reflected in numerous studies showing that women report higher levels of stress, anxiety, depression, and psychosomatic symptoms than men [3,4,5]. The increased prevalence of psychosomatic conditions among women [6] has amplified interest in holistic health approaches that address the interaction between psychological well-being and physiological processes [7]. In this context, the positive effects of psychological techniques such as stress management, mental relaxation, and guided imagery on health are being examined more closely [8,9,10].

The reciprocal interaction between the mind and body serves as a fundamental basis for alternative and complementary medicine approaches [11]. In recent years, mental techniques such as meditation, breathing exercises, yoga, guided imagery, and mindfulness-based practices have been widely used to promote psychological relaxation and regulate physiological systems [12,13]. Scientific research has shown these methods to provide various physiological benefits, such as enhancing immune system activity, supporting pain management, contributing to hormonal balance, and reducing stress responses [14,15,16]. The biopsychosocial processes that women experience throughout their life cycle—such as menstruation, pregnancy, childbirth, and menopause—make them particularly receptive to these mind–body techniques that activate their inner resources [17]. Therefore, mind–body practices specifically for women have become a prominent focus within holistic and integrative health care [18,19].

Guided imagery (GI) is a structured method that utilizes both meditation and relaxation techniques to guide an individual in harnessing the mind’s powerful capacity to influence bodily processes through the mental visualization of specific images, sounds, sensations, or experiences [20,21]. In practice, an individual is guided, often by a facilitator or an audio recording, to relax in a safe environment and mentally create an inner landscape, a healing experience, or a symbolic process [22]. Research has consistently shown that this method is effective in reducing stress levels [12,20], increasing pain tolerance [14], and accelerating pre- and post-operative recovery [23].

GI has been proven effective for various challenges women encounter throughout their life cycle. The method has been shown to reduce symptoms such as pain and tension during the menstrual cycle [24] and the anxiety and depression seen in premenstrual dysphoric disorder [25]. Furthermore, during pregnancy and childbirth, GI has been reported to alleviate birth anxiety and pre-cesarean fear [26,27,28], improve the overall birth experience [29], enhance quality of life in high-risk situations [30], and be capable of slowing the increase in blood pressure [31]. GI has been proven effective in various areas of women’s health, such as menopause, surgical preparation, chronic pain, and cancer care. The method has been reported to reduce stress and symptoms during the menopausal period [32], anxiety before gynecological surgery [33], and chronic pain conditions such as interstitial cystitis [34]. Furthermore, in cancer care, GI enhances emotional well-being and quality of life in breast cancer patients [35,36] and reduces anxiety in women with cervical cancer when delivered via virtual reality [37].

GI offers strong psychological and physiological support for women in coping with the biological and emotional changes they encounter throughout different stages of life, with studies demonstrating its effectiveness in enhancing comfort, reducing labor pain, and shortening the duration of labor [38,39,40]. Although previous studies have evaluated relaxation techniques or mind–body therapies, they remain fragmented and largely confined to isolated conditions—such as preoperative anxiety, specific breast cancer treatments, or isolated gestational milestones—failing to capture the broader, continuous trajectory of female health. Consequently, a critical literature gap persists regarding a dedicated, comprehensive synthesis that exclusively isolates pure GI interventions and evaluates their simultaneous psychophysiological outcomes across the entire spectrum of women’s life stages.

To address this gap, the main research question of this study is “What are the psychophysiological outcomes of guided imagery interventions across different life stages of women’s health?” Motivated by this question, this study aims to systematically evaluate randomized controlled trials examining these effects on women’s health. By mapping and synthesizing the findings across diverse female-specific health contexts, this review bridges the longstanding divide in clinical evidence, offering a unified, life-course perspective to guide future research and holistic nursing practice.

## 2. Materials and Methods

### 2.1. Protocol and Registration

The present study is a systematic review focusing on experimental studies. The conduct and reporting of the research were performed in accordance with the requirements of the PRISMA Statement, as published by Page et al. [41]. The review protocol was prospectively registered on 21 August 2025 in the PROSPERO database (registration number: CRD420251111577) to ensure methodological transparency and prevent duplication of efforts. To minimize bias, the literature screening, study selection, data extraction, and methodological quality appraisal were independently performed by two reviewers (S.S., D.G.). Full consensus at each stage was achieved through periodic meetings involving all three authors. Furthermore, a pilot was conducted with the participation of all three researchers to test key procedures, including keyword searching (on PubMed), article selection, data extraction, and quality assessment, thereby ensuring the robustness of the methodology.

### 2.2. Eligibility Criteria

The PICOS (Participants, Interventions, Comparisons, Outcomes, Study Design) framework was used to define the scope of the research question [42]. This review focused on experimental and quasi-experimental studies investigating the effects of guided imagery on the physical and psychological health of women. Specifically, the inclusion criteria encompassed studies conducted on female subjects experiencing physical and psychological health issues, utilizing simple guided imagery as the primary intervention, and comparing the intervention group with standard care, usual care, routine care, or no intervention (including multi-arm studies with a separate standard care control). The effectiveness of this intervention was evaluated by measuring outcomes such as anxiety, stress, depression, quality of life, pain, and fatigue. Studies published in English or Turkish without date restrictions were deemed eligible. Conversely, the exclusion criteria ruled out non-human studies, pediatric or adolescent populations unless the condition was specific to women’s health (e.g., primary dysmenorrhea), observational studies (cohort, case–control, cross-sectional, case series, and case reports), qualitative studies, reviews (systematic reviews, meta-analyses, and narrative reviews), non-research publications (editorials, commentaries, letters, protocols, and conference abstracts), studies lacking a control group (e.g., single-group pre-test/post-test designs), studies comparing only different variations of guided imagery without a standard care control, pharmacological interventions, publications with unclear methodology, and those with unavailable full texts.

### 2.3. Search Strategy

A comprehensive search of electronic databases was undertaken between 9 July and 21 July 2025 to identify relevant literature. The databases queried were PubMed (National Center for Biotechnology Information, Bethesda, MD, USA), EBSCO (EBSCO Information Services, Ipswich, MA, USA (Medline, CINAHL), Embase (Elsevier, Amsterdam, The Netherlands) (Ovid, Wolters Kluwer, Alphen aan den Rijn, The Netherlands), Web of Science (Clarivate, London, UK), PsycINFO (American Psychological Association, Washington, DC, USA), Scopus (Elsevier, Amsterdam, The Netherlands), and Cochrane (John Wiley & Sons, Inc., Hoboken, NJ, USA). In addition to the database search, the bibliographies of all eligible studies were manually screened to identify any other potentially relevant publications.

The search strategy employed a combination of medical subject headings (MeSH) and free-text keywords tailored to each database. The search string utilized the following core concepts combined with Boolean operators: (“women” OR “female” OR “woman”) AND (“guided imagery” OR “guided visualization” OR “imagery therapy” OR “mental imagery” OR “visualization therapy” OR “imagery-based intervention”) AND (“psychological well-being” OR “mental health” OR “anxiety” OR “depression” OR “stress” OR “emotional health” OR “mood” OR “quality of life” OR “pain management” OR “fatigue” OR “physiological response”). Following the primary database search and the removal of duplicates, reference lists of all included studies were meticulously screened for potential eligibility.

### 2.4. Study Selection

**Figure 1:**
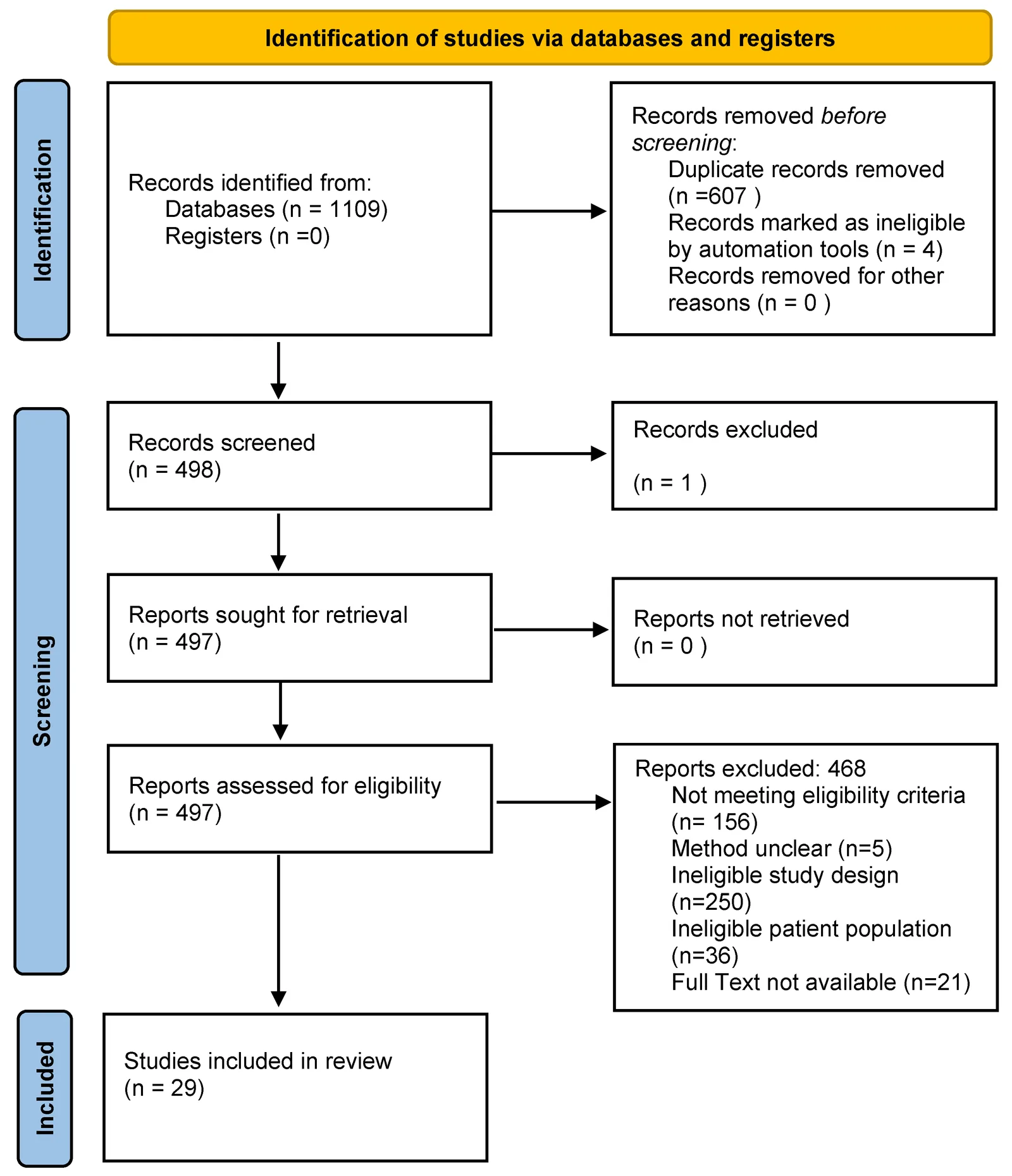
PRISMA flow diagram.

The reference management and article screening processes were systematically conducted using specialized software. Initially, all references identified across the databases were imported into EndNote, and duplicate records were removed. Subsequently, two independent reviewers screened the potential articles on the Covidence platform through the title, abstract, and full-text review stages. Pre-defined eligibility criteria were strictly followed throughout this screening process, and any disagreements were resolved through discussion and consensus within the research team. The entire article selection process is visualized in the PRISMA 2020 [41] flow diagram (Figure 1). The completed PRISMA 2020 checklist is provided in Supplementary Table S1.

### 2.5. Data Extraction

Systematic data extraction was performed for each included study using a meticulously designed standardized form. This template was structured to capture key information, including bibliographic data (author, year), study characteristics (design, setting, objective, sample size), participant details (study and comparison groups), intervention characteristics, and main outcomes as reported in the original studies. To minimize errors and bias, data abstraction was independently conducted by two reviewers, followed by a joint verification step to resolve any discrepancies (Table 1).

### 2.6. Methodological Quality Assessment of Studies

To assess the risk of bias and methodological quality, we employed the relevant Critical Appraisal Tools from the Joanna Briggs Institute. Depending on the study design, either the 13-item checklist for randomized controlled trials or the 9-item checklist for quasi-experimental studies was applied [56]. The appraisal was initially carried out in duplicate by two independent reviewers (the first and second authors). Subsequently, a consensus meeting was held with a third researcher to reconcile any disagreements and finalize the assessment for each study. The final quality score for each article was then used to categorize it as having “good” (80–100%), “fair” (50–79%), or “low” (<50%) quality, based on the framework by Goldsmith et al. [57].

### 2.7. Data Analysis

A narrative synthesis approach was used for the synthesis of the findings from the included studies. A statistical combination of the findings through meta-analysis was not deemed appropriate due to significant methodological, clinical, and statistical heterogeneity across the studies. Accordingly, the extracted data (including participant characteristics, intervention details, outcomes, and quality assessments) were first presented in summary tables. The findings were then grouped according to key characteristics such as intervention type, comparison groups, and outcomes measured. Based on these groupings, common patterns, consistencies, and contradictions in the evidence were analyzed and interpreted descriptively.

## 3. Results

### 3.1. Search Results

A total of 1109 records were identified through the database searches. After removing 607 duplicate records and 4 records marked as ineligible by automation tools, 498 remaining records were screened by title and abstract, resulting in the exclusion of 1 record. Subsequently, 497 full-text reports were sought, retrieved, and assessed for eligibility against the predefined criteria. Of these, 468 reports were excluded due to being irrelevant (*n* = 156), having an unclear method (*n* = 5), utilizing an ineligible study design (*n* = 250), involving an ineligible patient population (*n* = 36), or having an unavailable full text (*n* = 21). Ultimately, 29 studies met all inclusion criteria and were included in the systematic review.

### 3.2. Characteristics of Studies

Of the 29 studies included in this research, 27 were randomized controlled trials and two were quasi-experimental studies. The studies were published between 2005 and 2025. Six of the studies were conducted in the USA [20,33,34,44,46,47], four in Türkiye [25,27,28,39], three in Greece [32,52,53], three in Iran [16,48,50], two in Brazil [37,51], two in Canada [31,36], and one each in New Zealand [35], Israel [43], the United Kingdom [45], Norway [29], India [30], Spain [49], Thailand [54], Switzerland [38], and South Korea [55]. The total sample size of the studies was 2010 (intervention groups: 1045 participants; control groups: 965 participants). The participants in the included studies were adult women, with some studies specifying age ranges such as 18–65 or 40–65 years.

### 3.3. Characteristics of Participants

The total number of participants in the included studies ranged from 20 to 197. The research focused on diverse populations within women’s health, which can be categorized into four main groups:

#### 3.3.1. Women with Gynecologic and Breast Cancer

A significant portion of the studies was conducted on women diagnosed with cancer. This group included women with primary, advanced, or non-metastatic breast cancer [35,36,43,45,46,47,49,51,53,55]. Additionally, patients diagnosed with gynecologic cancers [49,52], such as cervical cancer [37], who were undergoing active treatments like brachytherapy, chemotherapy, or radiotherapy, were also part of the study samples.

#### 3.3.2. Women in Pregnancy, Childbirth, and Postpartum Periods

Research in this category focused on various stages of pregnancy and childbirth. The samples included women with high-risk pregnancies [27], pregnancy-induced hypertension [30,31], or those at risk of preterm birth [54]. Furthermore, pregnant women scheduled for cesarean sections [28], those in the active phase of labor [39], and healthy women in their second or third trimesters [16,20,29,38,48] were also studied.

#### 3.3.3. Women with Gynecological Conditions or Undergoing Surgery

This area of research involved women with specific gynecological diagnoses or those scheduled for surgical procedures. Participants included women diagnosed with conditions such as premenstrual dysphoric disorder (PMDD) [25] and interstitial cystitis (IC) [34]. The samples also comprised patients scheduled for colposcopy due to abnormal Pap smear results [44], those undergoing vaginal or pelvic floor surgery [33], and women experiencing sexual dysfunction [50].

#### 3.3.4. Menopausal Women

A selection of studies focused on peri- and postmenopausal women between the ages of 40 and 65 who were experiencing climacteric and stress-related symptoms [32].

### 3.4. Characteristics of Intervention

The interventions in the studies included in this review were primarily based on the guided imagery method, although they showed significant differences in terms of composition, delivery, and dosage. In many studies, GI was presented as a standalone intervention, typically via an audio recording, to manage issues such as preoperative anxiety [28], pelvic pain [34], or the stress of high-risk pregnancy [27]. Frequently, GI was combined with other relaxation methods, most commonly with progressive muscle relaxation [16,25,32,45,53,55], to reduce stress, anxiety, and the side effects of chemotherapy with virtual reality [37].

In some studies, GI was a core component of broader, multi-faceted programs, such as comprehensive coping strategies that taught cognitive restructuring [32,46], education program [32,35,46,54], and stress management programs for women undergoing cancer treatment [43,51,52,53]. Several studies used a design that compared the effectiveness of GI with other therapeutic methods, such as cognitive-behavioral therapy [43], foot reflexology [39], music [44], and massage therapy [48]. A few studies also specifically investigated guided imagery and music, a distinct psychotherapeutic method, to assess its impact on outcomes such as sexual function [50] or hope and fatigue in cancer patients [52]. These interventions, often delivered via customized audio recordings like CDs or tapes, were applied across a wide range of contexts, including for symptom management of various health conditions, during pregnancy, and for childbirth [20,29,33,34,47].

### 3.5. Quality Assessment Results

Following the assessment, it was observed that the quality scores of the randomized controlled trials (*n* = 27) ranged widely from 46% to 92%. The most frequently identified methodological shortcomings in these studies were the lack of blinding of participants, practitioners, and outcome assessors; unclear allocation concealment; and the failure to apply the intention-to-treat principle in the analyses. Conversely, the two quasi-experimental studies evaluated were found to possess high methodological quality, meeting all the criteria on the JBI checklist. Despite the variations in methodological scoring, all evaluated studies were deemed to be of sufficient quality for inclusion in this review. (Table 2).

### 3.6. Data Collection Tools

In the studies included in the review, a variety of validated measurement tools were used to assess the multifaceted effects of guided imagery interventions. Among the psychological variables, anxiety was the most frequently examined, largely measured by the State-Trait Anxiety Inventory [20,28,29,35,37,38,44,46,51] and its sub-forms. To assess anxiety and depression concurrently, the Hospital Anxiety and Depression Scale [25,49,53] was a commonly preferred tool, while the Perceived Stress Scale [20,27,54] was widely used to determine general stress levels. The Visual Analog Scale [38,39,44,49,52] was frequently utilized to measure the intensity of subjective symptoms such as pain and fatigue. Quality of life was examined as a significant outcome variable, especially in cancer populations, using cancer-specific scales like the Functional Assessment of Cancer Therapy [35,36,55] and the European Organization for Research and Treatment of Cancer Quality of Life Questionnaire [49]. In addition, more specific scales were also employed, such as the Beck Depression Inventory [29,46,51] for depression, the Pittsburgh Sleep Quality Index [32] for sleep quality, and the Profile of Mood States [47,52] to assess mood.

### 3.7. Outcomes

An analysis of the outcomes from the included studies provides a direct answer to our research question regarding the psychophysiological effects of guided imagery across women’s life stages, revealing consistent and positive impacts across various areas of women’s health. These results can be grouped under the main themes of reduction of psychological distress, management of pain and physical symptoms, improvement in quality of life, and enhancement of the birth experience (Table 3).

#### 3.7.1. Reduction of Psychological Distress (Anxiety, Stress, and Depression)

The most consistent finding across the studies is the positive impact of the interventions on psychological conditions such as anxiety, stress, and depression. The effectiveness of the method in reducing anxiety before and during surgical and medical procedures is particularly evident. For instance, studies demonstrated that guided imagery administered before a cesarean section reduced preoperative anxiety and surgical fear [28], while similar benefits were reported for women undergoing colposcopy [44] and patients undergoing brachytherapy [49]. There is also strong evidence for stress and anxiety reduction during pregnancy, including high-risk pregnancies [27], general courses of pregnancy [16,20], nulliparous pregnant women [48], and pregnant women at risk of preterm birth [54], where coping behaviors were also supported. Similar positive results regarding psychological distress were obtained in breast cancer patients [35,51], as well as in patients with cervical cancer when the method was delivered via virtual reality [37]. Finally, research involving women with premenstrual dysphoric disorder [25] and menopausal women [32] revealed that the interventions successfully reduced anxiety, depression, and stress.

#### 3.7.2. Management of Pain and Physical Symptoms

The interventions were also found to be effective in managing pain and other physical symptoms. For instance, guided imagery administered during labor reduced labor pain [39], and alleviation of pelvic pain [34] was reported in women diagnosed with interstitial cystitis. Positive results were also achieved for side effects related to cancer treatment, such as a significant reduction in chemotherapy-induced nausea and vomiting both in the hospital and at home [55], as well as improvements in fatigue and sleep problems [43,47] in breast cancer patients. Effects on physiological parameters were also observed, including lowered heart rate and stress hormones [38], alongside a slower increase in blood pressure in hypertensive pregnant women [31].

#### 3.7.3. Improvement of Quality of Life, Well-Being, and Birth Experience

The interventions were also emphasized by many studies to improve overall quality of life and psychological well-being. Improvements in quality of life were demonstrated in women with PMDD [25] as well as in breast cancer patients [36,45,46,53]. Furthermore, antepartum quality of life was improved in women with pregnancy-induced hypertension [30]. Specific enhancements were also reported, such as increased hope and reduced fatigue in cancer patients [52], alongside improvements in sexual dysfunction [50]. Positive effects on the birth experience were noted, including a positive impact on the birth experience itself [29], shortened labor duration, and increased birth satisfaction [39]. Finally, the method helped patients feel more prepared for surgery [33].

#### 3.7.4. Comparative Efficacy

Some studies compared the effectiveness of guided imagery with other methods. Research found the method to be as effective as cognitive-behavioral therapy, and in some areas, such as fatigue, even superior [43]. Furthermore, evidence demonstrated that guided imagery was more effective than progressive muscle relaxation in increasing relaxation levels [38]. Additionally, studies reported that both massage therapy and guided imagery reduced anxiety, though the acceptance rate for massage was higher [48].

## 4. Discussion

Synthesizing evidence from twenty-nine studies to address our research question regarding the psychophysiological impacts of guided imagery across women’s life stages, this systematic review reveals that the guided imagery method—both as a standalone intervention and as a component of multi-component programs—is a remarkably effective therapeutic tool. The findings directly answer this inquiry by demonstrating that the method provides consistent and significant benefits in areas such as reducing psychological distress, managing physical symptoms and pain, and enhancing overall quality of life and well-being.

As a non-pharmacological method, the effectiveness of guided imagery across such a wide range of women-specific health issues presents a new and valuable approach for clinical practice. This suggests that using the method as a complementary or alternative option to standard treatments holds the potential to create positive effects on women’s health and contribute holistically to patient care. However, the discrepancy between the method’s proven effectiveness and the fact that the number of studies in the literature and its prevalence in clinical practice are not yet at the desired level is a significant shortcoming. This situation clearly highlights the necessity for health professionals to conduct more evidence-based research in this area and to integrate the method more systematically into clinical practice.

A systematic review and meta-analysis synthesizing 21 studies on preoperative anxiety and postoperative pain has established the significance of the guided imagery method [23]. According to the results of this review, when administered by nurses, this method reduces preoperative anxiety and postoperative acute pain, while also being a suitable, easy, and low-cost intervention for both children and adults [23]. Supporting this finding, studies included in our review from [28] found that guided imagery administered before a cesarean section reduced preoperative anxiety and surgical fear; similarly, studies reported a decrease in anxiety in women undergoing colposcopy [44]. The positive effect of the method on pain is not limited to the surgical field. Indeed, studies also included in our review, stated that guided imagery administered during labor reduced labor contractions and pain [39], while other studies reported an alleviation of pelvic pain in women diagnosed with interstitial cystitis [34]. Collectively, these findings demonstrate that guided imagery is an effective, non-invasive, and widely nurse-applicable method for managing both anxiety and pain in various clinical situations such as surgery, gynecological procedures, and labor.

Although a previous meta-analysis on the combined use of progressive muscle relaxation and guided imagery in cancer patients emphasized psychological benefits while noting a lack of definitive evidence regarding physical side effects [58], our systematic review addresses this gap by synthesizing findings across diverse oncological populations (e.g., patients undergoing chemotherapy or palliative care) and varying intervention dosages (typically ranging from 2 to 6 weeks with sessions lasting 15 to 30 min). Our findings demonstrate with a moderate level of certainty that guided imagery—even when applied as a standalone intervention—not only alleviates psychological distress, anxiety, and depression [35,37,49,51] and fosters hope [52], but also significantly mitigates chemotherapy-induced nausea and vomiting [55], fatigue, and sleep disturbances [43,47,52]. These results align with broader clinical trials showing that structured guided imagery protocols significantly outperform control conditions in reducing both psychological symptoms and physical side effects such as pain, insomnia, and appetite loss [59]. Consequently, by explicitly defining population parameters and dosage conditions, our findings substantiate that guided imagery is a robust, evidence-based adjunct intervention capable of managing both psychological and physical burdens in cancer care, thereby supporting its targeted integration into standard oncological protocols.

Guided imagery is widely utilized to mitigate stress, anxiety, elevated blood pressure, and pain across the prenatal and postpartum continuum [21]. Supporting broader literature—such as a comprehensive meta-analysis of 32 studies identifying relaxation techniques as promising for maternal-infant health [60]—our review synthesizes findings from pregnant populations across varying gestation periods and structured intervention regimens (typically consisting of daily or weekly audio-guided sessions over 4 to 12 weeks). With a moderate level of certainty, our review demonstrates that these techniques yield targeted physiological and psychological benefits: during pregnancy, they significantly reduce maternal anxiety and stress [16,20,27,48,54] and modulate autonomic markers such as heart rate, cortisol/stress hormones, and blood pressure [31,38]; during active labor, they optimize the birth experience, shorten labor duration, and enhance maternal satisfaction [29,39]. Furthermore, these benefits extend directly to neonatal outcomes; specific clinical trials in high-risk populations, such as women diagnosed with preterm labor, indicate that consistent daily guided imagery applications significantly lower rates of low birth weight and neonatal intensive care unit (NICU) admissions [61]. Collectively, by clearly specifying population risk levels, gestational timing, and intervention dosages, this evidence establishes a rigorous rationale for integrating guided imagery as a standardized, non-pharmacological component of comprehensive perinatal care.

In summary, the evidence synthesized in this review demonstrates that guided imagery functions as a versatile and potent non-pharmacological intervention across various vulnerable populations, particularly in managing psychological distress, pain, and physical side effects. However, critical examination of the current literature reveals that clinical heterogeneity—stemming from varying intervention dosages, session durations, delivery formats, and patient baseline characteristics—limits the generalizability of certain findings. While our synthesis indicates that standardized protocols (such as structured multi-week audio sessions) yield reliable benefits, future research must prioritize rigorous, large-scale randomized controlled trials with precise dosage parameters and long-term follow-ups to definitively establish its efficacy. Addressing these methodological gaps will be essential for safely and systematically translating guided imagery from research settings into mainstream, evidence-based nursing and clinical care.

## 5. Conclusions

In conclusion, addressing the core research question regarding the psychophysiological outcomes of guided imagery across women’s life stages, this systematic review establishes that the method serves as a holistic and versatile intervention—spanning from oncology to perinatal care, surgery, and pain management—that improves both psychological and physiological outcomes. The evidence presented confirms the method’s power to alleviate psychological challenges like anxiety, stress, and depression, while also strongly demonstrating its effectiveness on concrete physical indicators such as pain, nausea, labor duration, and newborn health. Therefore, bridging the gap between these evidence-based findings and current clinical practice is of critical importance. As a low-cost, non-invasive, and patient-centered approach, guided imagery is a valuable therapeutic tool with high potential to become a standard component of women’s health services.

## Figures and Tables

**Table 1:** The list of studies included in the systematic review.

Author(s), Year, Country	Study Design, Year	Objective	Data Collection Tools	Sample Characteristics	Characteristics of the Intervention	Main Outcomes
Abaoğlu, Çiftçi & Ekici, 2024, Türkiye [25]	RCT, June 2019 and October 2021	To examine the effectiveness of relaxation training in women with premenstrual dysphoric disorder.	PSST, HADS, NHP, SDS	I: 30, C: 33, women who met the DSM 5 criteria for PMDD	Relaxation training including progressive muscle relaxation and guided imagination	Relaxation training had a positive effect on reducing premenstrual symptoms, anxiety, depression and disability and improving quality of life in women with PMDD.
Aker, Öner Cengiz & Yılmaz Sezer, 2024, Türkiye [28]	RCT, March and May 2023	To investigate the impact of guided imagery applied before cesarean section on preoperative anxiety, surgical fear, and physiological parameters.	STAI-S, SFQ	I: 29, C: 28, pregnant women who underwent cesarean section surgery	15 min guided imagery audio recording three days before their cesarean section surgery	The guided imagery intervention before cesarean section was effective in reducing preoperative anxiety and keeping surgical fear under control.
Augoulea et al., 2021, Greece [32]	RCT, 2015– 2017	To evaluate the effectiveness of a lifestyle education program incorporating relaxation techniques (deep breathing, progressive muscle relaxation, and guided visualization) on climacteric symptoms in peri- and postmenopausal women.	GCS, PSQI, DASS-21, RSS, HLC	I: 31, C: 30, women aged 40–65 years with varying climacteric and stress symptoms	Stress management program for 8 weeks, which consisted of one weekly session on the benefits of regular exercise, healthy diet, cognitive restructuring, and learning relaxation techniques such as diaphragmatic breathing, progressive neuromuscular relaxation, and guided visualization.	The intervention significantly reduced both stress and climacteric symptoms.
Billquist et al., 2017, USA [33]	RCT, from July 2014 to October 2015	To determine if preoperative guided imagery would help women to feel more prepared, less anxious, and have higher satisfaction scores 6 weeks after vaginal or pelvic floor surgery.	PFDI, POPQ, STAI	I: 18, C: 20, women scheduled for vaginal or abdominal pelvic floor surgery	The guided imagery group was shown a customized CD that explained the events and expectations of the surgery day using both guided imagery meditation and relaxation techniques.	The implementation of self-administered guided imagery meditation was associated with enhanced patient preparedness for pelvic floor surgery necessitating an overnight hospital stay.
Cameron et al., 2007, New Zealand [35]	Quasi- experimental, June 2000 and March 2003	To assess the efficacy of a group intervention in altering emotion regulation processes and promoting adjustment in women with breast cancer.	CECS, IPQ-R, BFS-BC, FACT, STAI-Short	I: 54, C: 44, women diagnosed with primary breast cancer within the past six weeks	Education program about emotion and cancer; training in relaxation, imagery, meditation, setting priorities and goals, emotional disclosure through writing, and anger management; and group discussion.	An emotion regulation-based intervention positively influenced psychological adjustment in women during the first year after a breast cancer diagnosis by enhancing emotional well-being and reducing anxiety and emotional suppression.
Carrico et al., 2007, USA [34]	RCT, from July 2005 to August 2006	To explore the effect of guided imagery on pelvic pain and urinary symptoms in women with interstitial cystitis (IC) symptoms.	IC-SIPI, GRA	I: 15, C: 15, women with a medical diagnosis of IC by a board-certified urologist.	Listened to a 25 min guided imagery CD (specifically created for IC and pelvic pain) twice daily for 8 weeks.	The guided imagery method contributes to pain reduction and shows a tendency toward symptom improvement when used for pain and symptom management in women with interstitial cystitis.
Cohen & Fried, 2007, Israel [43]	RCT	To assess the effectiveness of cognitive-behavior (CB) group intervention versus relaxation and guided imagery (RGI) group training in women with breast cancer.	BSI, GSI, FSI, MSQ, MHLC	CB: 38, RGI: 39, C: 37, women diagnosed with breast cancer.	CB group intervention versus relaxation and guided imagery RGI group training.	Both RGI and CB interventions are effective in reducing psychological distress in breast cancer patients, with RGI being more effective in reducing fatigue and sleep problems.
Dal, Beydağ & Doğan, 2023, Türkiye [27]	RCT, February–May 2021	To identify the effect of the practice of guided imagery on the perceived stress level in high-risk pregnancies.	PSS	I: 64, C: 64, women who had high-risk pregnancies	Guided imagery on the perceived stress level in high-risk pregnancies.	Guided imagery reduced perceived stress in women with high-risk pregnancies.
Danhauer et al., 2007, USA [44]	RCT	To evaluate the effectiveness of music or guided imagery in reducing anxiety and perceived pain compared to usual care in patients undergoing colposcopy.	STAI, VAS, Patient Satisfaction Questions	M: 56, GI: 56, C: 58, women scheduled for colposcopy due to an abnormal Pap smear.	Music and guided imagery	The benefits of music and guided imagery interventions to decrease anxiety and perceived pain for women undergoing colposcopy.
Eremin et al., 2009, United Kingdom [45]	RCT	To evaluate the immunological effects of relaxation and guided imagery in women undergoing multimodality treatment for locally advanced breast cancer (LABC).	Immunological assays	I: 40, C: 39, women under 75 years of age with large (>4 cm) or LABC (T3, T4, Tx N2)	Relaxation and guided imagery training	Relaxation and guided imagery training improved quality of life, enhanced mood, and strengthened emotional expression in women undergoing treatment for advanced-stage breast cancer.
Esplen et al., 2018, Canada [36]	RCT	To assess the efficacy of an 8-week group intervention in women after breast cancer treatment.	FSFI, FACT–Breast, BIS	I: 131, C: 63, women who had been diagnosed with non-metastatic invasive breast cancer and had completed adjuvant radiotherapy and chemotherapy.	A manual-based intervention incorporating expressive guided imagery exercises integrated with principles of group therapy.	The group intervention in this study improved the quality of life for breast cancer survivors but was not effective in addressing sexual dysfunction.
Gaston-Johansson et al., 2013, USA [46]	RCT	The purpose of this study was to examine the impact of the self-management comprehensive coping strategy program (CCSP) on quality of life among women with breast cancer 1 year after treatment.	QOLI-CV, STAI, BDI,	I: 52, C: 58, Women are diagnosed with advanced breast cancer.	The CCSP intervention was taught two weeks before hospital admission and reinforced during treatment and three months after discharge, including education, cognitive restructuring, coping skills enhancement, and relaxation with guided imagery.	The CCSP improved quality of life for breast cancer patients at the 1-year follow-up and was widely reported as beneficial, showing potential as an effective self-management coping intervention for survivors.
Gedde-Dahl & Fors, 2012, Norway [29]	RCT	The objective of this study was to test if and how self-administered practice of relaxation techniques, positive affirmation and guided imagery.	STAI-S/T, BDIe, VAS	I: 27, C: 27, Pregnant women in the early third trimester	A CD containing relaxation techniques, positive affirmations, and a guided imagery technique.	The relaxation and guided imagery techniques, which healthy pregnant women can practice on their own, positively affect the birth experience.
Goodwin et al., 2006, USA [47]	RCT	The aim of the study was to evaluate the effectiveness of guided imagery and relaxation interventions to enhance psychological well-being in women with breast cancer.	POMS	I: 26, C: 26, Women with Stage I and Stage II breast cancer	The intervention consisted of participants listening to a guided imagery or relaxation tape five times a week for four weeks, supported by weekly follow-up phone calls.	The guided imagery group showed significant improvements in depression, anxiety, and fatigue, while no changes were reported in the relaxation group.
Jallo et al., 2014, USA [20]	RCT	The purpose of this study was to evaluate the efficacy of a guided imagery intervention for stress reduction in pregnant African American women beginning early in the second trimester.	PSS, NRSS, STAI, BFI	I: 36, C: 36, African American pregnant women between 14 and 17 weeks of gestation	The guided imagery method was delivered over a 12-week period through a CD containing four professionally recorded audio tracks.	The guided imagery intervention was effective in reducing perceived stress, anxiety, and fatigue at week 8 among pregnant African American women; however, these effects did not persist at week 12. No significant differences were observed between groups in Corticotrophin Releasing Hormone levels.
Jesy et al., 2025, India [30]	RCT	This study evaluates the effectiveness of guided imagery therapy in enhancing the quality of life among women with Pregnancy Induced Hypertension.	QoLS	I: 30, C: 30, women diagnosed with PIH.	Guided imagery therapy, consisting of 10 sessions over 4 weeks, in addition to standard medical care.	Guided imagery therapy effectively enhances antepartum quality of life in women with PIH, supporting its integration as a complementary intervention in maternal care.
Kaplan & Çevik, 2021, Türkiye [39]	RCT, 15 June 2019 and 15 March 2020	To determine the effect of guided imagery and foot reflexology on pain intensity, duration of labor, and birth satisfaction during labor.	VAS, BSS	GI: 40, FR: 40, C: 40, Pregnant women with a cervical dilatation of 4 cm.	The guided imagery group has watched the guided imagery video; the reflexology group has received reflexology for both feet for 30 min.	The experimental groups experienced less labor pain compared to the control group, and reflexology and guided imagery practices shortened the active, transitional, and second stages of labor while increasing birth satisfaction.
Khojasteh et al., 2016, Iran [48]	RCT, 21 January 2016–19 May 2016	To compare the effects of massage therapy and guided imagery on anxiety during pregnancy in nulliparous women.	PRAQR	MT: 25, GI: 25, C: 25, nulliparous women during pregnancy (gestational weeks 22 to 28).	The intervention consisted of Massage Therapy and guided imagery, compared to a control group.	Both massage therapy and guided imagery reduced anxiety during pregnancy; however, massage therapy was more widely accepted by all women, while guided imagery was preferred more by those with higher education levels.
Leo ’n-Pizarro et al., 2007, Spain [49]	RCT, from May 2002 to November 2003	To determine the degree of anxiety and depression in patients with breast or gynecological cancer during brachytherapy treatment and evaluate the effects of psychological intervention consisting of in training in relaxation techniques and guided imagery.	HADS, CCV, VAS	I: 47, C: 47, women with breast or gynecological cancer.	In both groups received training on brachytherapy, but only the study group patients received training in relaxation and guided imagery.	The use of relaxation techniques and guided imagery is effective in reducing the levels of anxiety, depression and body discomfort in patients who must remain isolated while undergoing brachytherapy.
Moffatt et al., 2010, Canada [31]	RCT	To provide preliminary evidence of whether relaxation by means of guided imagery would reduce blood pressure in hypertensive pregnant women.	Y-1	I: 34, C: 35, pregnant women with hypertension	Guided imagery or of quiet rest, twice daily for 4 weeks or until delivery.	No statistically significant reduction in mean arterial pressure was observed following the guided imagery intervention; however, the increase in blood pressure was lower in the guided imagery group compared to the quiet rest group.
Mohammadi et al., 2023, Iran [50]	RCT, from 20 November 2018 to 15 August 2019	To determine the effect of guided imagery and music on the level of sexual function in women with sexual dysfunction.	FSFI	I: 36, C: 36, women with sexual dysfunction	GIM in which the text and music	Guided imagery and music as an effective, easy, affordable, and usable method in Iranian culture can be effective in improving the sexual function of reproductive-age women.
Nasiri et al., 2018, Iran [16]	RCT, from September 2013 to November 2014	The aim of measuring the effect of progressive muscle relaxation and guided imagery on stress, anxiety, and depression in pregnant women.	EDS, DASS-21	I: 33, C: 33, pregnant women at 28–36 weeks.	Progressive muscle relaxation and guided imagery techniques.	Relaxation and guided imagery were found to reduce stress, anxiety, and depression in pregnant women over six sessions.
Nunes et al., 2007, Brazil [51]	RCT	The effects of relaxation and visualization therapy on psychological distress, cortisol levels, and immunological parameters of breast cancer patients undergoing radiotherapy.	STAI, BAI, BDI	I: 20, C: 14, women with breast cancer (Stage I or II) undergoing radiotherapy.	The RVT intervention was implemented over 24 days through daily structured group sessions involving relaxation and guided imagery techniques.	This study adds to the growing literature suggesting that RVT can effectively attenuate psychological distress and may thus improve the quality of life of cancer patients undergoing radiotherapy.
Papanikolaou et al., 2023, Greece [52]	RCT	Investigate the potential effectiveness of the music psychotherapy method, guided imagery and music, to assist female patients who are undergoing chemotherapy treatment for breast or gynecologic cancer.	POMS-Brief, CFS, HHI, VAS-Hope; VAS-Fatigue	I: 10, C: 10, Female patients receiving treatment for breast or gynecologic cancer at a gynecologic oncology unit in Athens.	Guided imagery and music sessions, each lasting up to 60 min, were conducted.	A brief series of GIM sessions shows promise to be beneficial for increasing hope, decreasing fatigue, and mitigating distressed mood for female patients undergoing treatment for breast or gynecologic cancer.
Santana et al., 2023, Brazil [37]	RCT, from October 2019 to January 2021	To evaluate the effect of guided imagery relaxation through virtual reality on anxiety in women with cervical cancer undergoing radio chemotherapy.	STAI, STAI-S	I: 28, C: 24, women with cervical cancer undergoing radio chemotherapy.	12 sessions of guided imagery relaxation through virtual reality, applied three times a week.	Guided imagery relaxation through virtual reality provided evidence of anxiety reduction in women with cervical cancer undergoing radio chemotherapy and may contribute to clinical practice.
Seliniotaki et al., 2021, Greece [53]	RCT	To investigate the effects of stress management program, carried out by one psychologist in women treated for breast cancer	EORTC QLQ-C30 version 3.0, HADS, PTGI, ERRI, SSGS	I: 27, C: 26, Female patients diagnosed with breast cancer, with an ECOG performance status of 0–2 and currently receiving active treatment.	In addition to the 8-week individual stress management program, patients were provided with audio recordings to practice either progressive muscle relaxation or guided imagery techniques twice daily.	The 8-week stress management program combined with guided imagery practices led to improvements in various physical and psychological aspects such as body mass index, quality of life, emotional and cognitive functioning in patients with breast cancer.
Ukhawounam et al., 2023, Thailand [54]	Quasi-experimental, April and December 2022	To determine the effects of education and guided imagery program on stress level and coping behaviors among pregnant women at risk of preterm birth.	PSS-10, Coping Behavior Questionnaire	I: 24, C: 24, pregnant women who visited the antenatal care unit were at risk of preterm birth with moderate stress score were included.	The education and guided imagery program were 5 weeks and conducted through LINE application, Google form, and telephone.	This research highlights the importance of preventing mental health problems in pregnant women at risk of preterm birth by using an education and guided imagery program to decrease stress and promote appropriate coping behaviors.
Urech et al., 2010, Switzerland [38]	RCT	To compare the immediate effects of two active and one passive 10 min relaxation technique on perceived and physiological indicators of relaxation with pregnant women.	VAS, STAI-S	Progressive muscle relaxation: 13, GI: 13, C: 13, pregnant women between the 32nd and 34th weeks of gestation	Pregnant women participated in a 10 min relaxation exercise using progressive muscle relaxation, guided imagery, or passive relaxation, with hormonal and physiological measurements taken before and after the intervention.	Guided imagery outperformed progressive muscle relaxation and controls in boosting relaxation, while both methods significantly reduced heart rate. Most relaxation techniques lowered stress hormones, excluding epinephrine, with guided imagery proving especially effective for pregnant women.
Yoo et al., 2005, South Korea [55]	RCT, early 2002–2004	To assess the effectiveness of progressive muscle relaxation training and guided imagery in reducing the anticipatory nausea and vomiting and post chemotherapy nausea and vomiting of patients with breast cancer and to measure their effects on the patients’ quality of life.	MAACL, FACT-Bv4	I: 30, C: 30, women newly diagnosed with primary breast cancer, who had undergone surgery within 1–2 months	Six progressive muscle relaxation training and guided imagery sessions administered by a therapist 1 h before each chemotherapy cycle at the hospital, along with a recorded tape of the first session for home practice at least 3 days post chemotherapy to manage delayed symptoms such as nausea, vomiting, and anxiety.	Patients who received PMRT and guided imagery showed a significant reduction in anticipatory nausea and vomiting and post-treatment nausea and vomiting both in the hospital and at home.

Abbreviations: BDI: Beck Depression Inventory, BDIe: Beck Depression Inventory (self-report format), BFS-BC: Benefit-Finding Scale for Breast Cancer, BFI: Brief Fatigue Inventory, BIS: Body Image Scale, BSI: Brief Symptom Inventory, BSS: Birth Satisfaction Scale, CCV: Cuestionario de Calidad de Vida QL-CA-AFex, CECS: Cortauld Emotional Control Scale, DASS-21: Depression Anxiety Stress Scale–21, EDS: Edinburgh Depression Scale, EORTC QLQ-C30 v3.0: European Organization for Research and Treatment of Cancer Quality of Life Questionnaire Version 3.0, ERRI: Event Related Rumination Inventory, FACT: Functional Assessment of Cancer Therapy, FACT-B: Functional Assessment of Cancer Therapy–Breast, FACT-Bv4: Functional Assessment of Cancer Therapy–Breast, Version 4, FSFI: Female Sexual Function Index, FSI: Fatigue Symptom Inventory, GCS: Green Climacteric Scale, GRA: Global Response Assessment, GSI: Global Severity Index, HADS: Hospital Anxiety and Depression Scale, HHI: Herth Hope Index, HLC: Health Locus of Control, IC-SIPI: Interstitial Cystitis Symptom Index and Problem Index, IPQ-R: Illness Perceptions Questionnaire-Revised, MAACL: Multiple Affect Adjective Checklist, MHLC: Multidimensional Health Locus of Control, MSQ: Mini Sleep Questionnaire, NHP: Nottingham Health Profile, NRSS: Numeric Rating Scale of Stress, PFDI: Pelvic Floor Distress Inventory, POPQ: Pelvic Organ Prolapse Quantification System, POMS: Profile of Mood States, POMS-Brief: Profile of Mood States–Brief, PRAQR: Pregnancy-Related Anxiety Questionnaire–Revised, PSQI: Pittsburgh Sleep Quality Index, PSS: Perceived Stress Scale, PSS-10: Perceived Stress Scale–10 items, PSST: Premenstrual Symptoms Screening Tool, PTGI: Post-Traumatic Growth Inventory, QOLI-CV: Quality of Life Index–Cancer Version, RSS: Rosenberg Self-Esteem Scale, SDS: Sheehan Disability Scale, SFQ: Surgical Fear Questionnaire, SSGS: State Shame and Guilt Scale, STAI: State-Trait Anxiety Inventory, STAI-Short: Spielberger State-Trait Anxiety Inventory–Short Form, STAI-S: State Anxiety Inventory–State Form, STAI-S/T: State-Trait Anxiety Inventory–State/Trait Forms, VAS: Visual Analog Scale, VAS-Fatigue: Visual Analogue Scale–Fatigue, VAS-Hope: Visual Analogue Scale–Hope, Y-1: State Scale of the State-Trait Anxiety Inventory.

**Table 2:** Quality assessment scores of the studies.

Studies	JBI Critical Appraisal Checklist Questions for Randomized Controlled Studies													Quality Score of the Study
	Q1	Q2	Q3	Q4	Q5	Q6	Q7	Q8	Q9	Q10	Q11	Q12	Q13	
Abaoğlu, Çiftçi & Ekici, 2024 [25]	Y	N	Y	N	N	N	Y	Y	Y	Y	Y	Y	Y	69.23%
Aker, Öner Cengiz & Yılmaz Sezer, 2024 [28]	Y	Y	Y	N	N	Y	Y	Y	N	Y	Y	Y	Y	76.92%
Augoulea et al., 2021 [32]	Y	N	Y	N	N	N	Y	Y	Y	Y	Y	Y	Y	69.23%
Billquist et al., 2017 [33]	Y	Y	Y	N	N	N	Y	Y	N	Y	Y	Y	Y	69.23%
Carrico et al., 2007 [34]	Y	Y	Y	N	N	N	Y	Y	N	Y	Y	Y	Y	69.23%
Cohen & Fried, 2007 [43]	Y	N/A	Y	N	N	Y	Y	Y	N	Y	Y	Y	Y	69.23%
Dal, Beydağ & Doğan, 2023 [27]	Y	Y	Y	N	N	Y	Y	Y	Y	Y	Y	Y	Y	84.61%
Danhauer et al., 2007 [44]	Y	N	Y	N	N	N	Y	Y	N	Y	Y	Y	Y	61.54%
Eremin et al., 2009 [45]	Y	Y	Y	N	N	Y	Y	Y	Y	Y	Y	Y	Y	84.61%
Esplen et al., 2018 [36]	Y	Y	N	N	N	N/A	Y	Y	N	Y	Y	Y	Y	61.54%
Gaston-Johansson et al., 2013 [46]	Y	N/A	Y	N	N	N	Y	Y	N	Y	Y	Y	Y	61.54%
Gedde-Dahl & Fors, 2012 [29]	Y	Y	N	N	Y	Y	Y	Y	N	Y	Y	Y	Y	76.92%
Goodwin et al., 2006 [47]	Y	N/A	N/A	N	N	N	Y	Y	N	Y	Y	Y	Y	53.85%
Jallo et al., 2014 [20]	Y	N/A	Y	N	N	N/A	Y	Y	Y	Y	Y	Y	Y	69.23%
Jesy et al., 2025 [30]	Y	N	Y	N	N	N/A	Y	N/A	Y	Y	Y	Y	Y	61.54%
Kaplan & Çevik, 2021 [39]	Y	N/A	Y	N	N	N	Y	Y	Y	Y	Y	Y	Y	69.23%
Khojasteh et al., 2016 [48]	Y	N/A	N/A	N	N	N/A	Y	Y	Y	Y	Y	Y	Y	61.54%
Leo ’n-Pizarro et al., 2007 [49]	Y	N/A	Y	N	N	N	Y	Y	Y	Y	Y	Y	Y	69.23%
Moffatt e al., 2010 [31]	Y	Y	Y	N	N	N	Y	Y	N	Y	Y	Y	Y	69.23%
Mohammadi et al., 2023 [50]	Y	Y	Y	N	N	Y	Y	Y	Y	Y	Y	Y	Y	84.61%
Nasiri et al., 2018 [16]	Y	Y	Y	N	N	Y	Y	Y	Y	Y	Y	Y	Y	84.61%
Nunes et al., 2007 [51]	Y	N/A	Y	N	N	Y	Y	Y	N	Y	Y	Y	Y	69.23%
Papanikolaou et al., 2023 [52]	Y	N/A	Y	N	N	N	Y	Y	Y	Y	Y	Y	Y	69.23%
Santana et al., 2023 [37]	Y	Y	Y	N	N	Y	Y	Y	Y	Y	Y	Y	Y	84.61%
Seliniotaki et al., 2021 [53]	Y	N/A	Y	N	N	N/A	Y	Y	Y	Y	Y	Y	Y	69.23%
Urech et al., 2010 [38]	Y	Y	Y	Y	Y	Y	Y	Y	N	Y	Y	Y	Y	92.31%
Yoo et al., 2005 [55]	Y	N/A	Y	N	N	N/A	Y	Y	N	Y	Y	Y	Y	61.54%
Question quality score	100%	44.4%	85.1%	3.7%	7.4%	37.3%	100%	96.9%	51.8%	100%	100%	100%	100%	
	JBI Critical Appraisal Checklist Questions for Quasi-Experimental Studies													
Cameron et al., 2007 [35]	Y	Y	Y	Y	Y	Y	Y	Y	Y					100%
Ukhawounam et al., 2023 [54]	Y	Y	Y	Y	Y	Y	Y	Y	Y					100%
Question quality score	100%	100%	100%	100%	100%	100%	100%	100	100%					

Abbreviations: Y: Yes, N: No, N/A: Not Applicable.

**Table 3:** Summary of included studies categorized by intervention type and measured outcomes.

Intervention Type	Target Population/Health Condition	Measured Primary Outcomes	Number of Studies & References
Audio/Cd/Vr-Assisted Guided Imagery	Pregnancy, Cesarean Section, Pelvic Floor Surgery, Interstitial Cystitis, Cervical Cancer	Anxiety, Surgical Fear, Stress, Pain, Sleep Quality, Quality of Life, Physiological Parameters (BP, Pulse)	13 Studies [20,27,28,29,30,31,33,34,37,38,39,44,54]
Guided Imagery and Music (GIM/Music Psychotherapy)	Breast or Gynecological Cancer, Sexual Dysfunction	Depression, Hope, Fatigue, Distressed Mood, Sexual Function	3 Studies [50,52,55]
Multicomponent Stress Management/Education Programs (Relaxation + Imagery + Cognitive Therapy)	Menopause, Breast Cancer, PMDD	Climacteric Symptoms, Psychological Distress, Emotional Expression, Quality of Life, Fatigue	9 Studies [25,32,35,36,43,45,46,47,53]
Relaxation Therapies and Visualization Combinations (PMR + Guided Imagery)	Pregnancy, Cancer	Anxiety, Depression, Stress, Cortisol/Immune Parameters, Nausea-Vomiting	4 Studies [16,48,49,51]

## Data Availability

No new data were created or analyzed in this study. Data sharing is not applicable to this article.

## Supplementary Materials

The following supporting information can be downloaded at: https://www.mdpi.com/article/10.3390/ijerph23081066/s1, Table S1: PRISMA 2020 checklist.
